# The toxicity outcome of silica nanoparticles (Ludox®) is influenced by testing techniques and treatment modalities

**DOI:** 10.1007/s00216-012-6246-6

**Published:** 2012-07-22

**Authors:** Caterina Fede, Francesco Selvestrel, Chiara Compagnin, Maddalena Mognato, Fabrizio Mancin, Elena Reddi, Lucia Celotti

**Affiliations:** 1 Department of Biology, University of Padova, via U. Bassi 58/B, 35131 Padova, Italy; 2 Department of Chemical Sciences, University of Padova, via Marzolo 1, 35131 Padova, Italy; 3 Laboratori Nazionali di Legnaro-INFN Legnaro, 35100 Padova, Italy

**Keywords:** Nanoparticles, Cell systems, Dynamic light scattering, MTS assay, Clonogenic assay

## Abstract

We analyzed the influence of the kind of cytotoxicity test and its application modality in defining the level of hazard of the in vitro exposures to nanostructures. We assessed the cytotoxicity induced by two different Ludox® silica nanoparticles (NPs), AS30 and SM30, on three human cell lines, CCD-34Lu, A549, and HT-1080. Dynamic light scattering measurements showed particle agglomeration when NPs are diluted in culture medium supplemented with fetal calf serum. We examined the impact of such particle aggregation on the cytotoxicity by exposing the cells to NPs under different treatment modalities: short incubation (2 h) in serum-free medium or long incubation (24–72 h) in serum-containing medium. Under this last modality, NP suspensions tended to form aggregates and were toxic at concentrations five- to tenfold higher than in serum-free medium. The results of cell survival varied considerably when the long-term clonogenic assay was performed to validate the data of the short-term MTS assay. Indeed, the half maximum effective concentrations (EC_50_) in all the three cell lines were four- to fivefold lower when calculated from the data of clonogenic assay than of MTS. Moreover, the mechanisms of NP toxicity were cell-type-specific, showing that CCD-34Lu are prone to the induction of plasma membrane damages and HT-1080 are prone to DNA double-strand break and apoptosis induction. Taken together, our results demonstrate that the choice of testing strategy and treatment conditions plays an important role in assessing the in vitro toxicity of NPs.

**Electronic supplementary material:**

The online version of this article (doi:10.1007/s00216-012-6246-6) contains supplementary material, which is available to authorized users.

**Figure:**
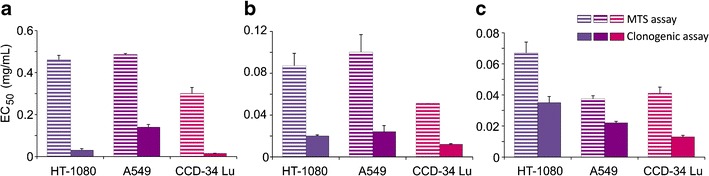

## Introduction

Nanoparticles (NPs) are particulate structures of various shapes and different compositions with a 1–100 nm size. These structures possess unique and innovative physical and chemical properties, determined by their nanoscale dimensions and especially by the high-ratio surface area/volume that give to the NPs a new chemical reactivity and new optical, magnetic, catalytic, and electrochemical properties. In the last decades, these characteristics have made the NPs of considerable interest in technological development and widely used in medicine and diagnostics [1], in biotechnology [2, 3], and in cosmetics, food, and materials [4]. Silica NPs (SiO_2_) have found extensive applications in industrial manufacturing, packaging, chemical industry, and as additives to drugs, cosmetics, printer toners, and food. In recent years, the use of silica nanoparticles has been extended to biomedical and biotechnological fields, such as biosensors or biomarkers for optical microscopy imaging [5], cancer therapy [6], DNA delivery [7, 8], and drug delivery [9].

However, the increasing exposure to nanoscale particles requires studies that characterize their properties and potential cytotoxic effects in order to provide exhaustive information for the assessment of the impact of nanomaterials on human health and the consequent regulation of their use. To date, several studies have shown the cytotoxicity of silica nanoparticles in vitro and in vivo. These reports demonstrated that exposure to SiO_2_ NPs can cause decrease of cell viability as a function of size, dose, and time of exposure [10–12] and in a surface area-dependent manner in human primary endothelial cells [13]. A size-, dose-, and time-dependent cytotoxicity related to oxidative stress has been observed in human cells exposed to SiO_2_ NPs [12, 14–17], together with oxidative stress-driven apoptosis [12, 18]. Silica NPs have the ability to induce inflammatory responses in cultured primary human pulmonary fibroblasts [19], in human endothelial cells [20] and in mouse macrophage cell line [21], as well as to induce cell cycle arrest in human myocardial and in embryonic kidney cells [11, 22]. In vivo exposure to SiO_2_ NPs caused hepatotoxicity [23], liver injury [24], pregnancy complications [25], increased level of pro-inflammatory cytokines in mice [21], and pulmonary and cardiovascular damage with ischemic disorders in old rats [26]. Moreover, silica nanoparticles that enter the nucleus induce the formation of protein aggregates, inhibiting DNA replication and transcription [27]. Along with size, dose, and incubation time, differences in cytotoxicity induced by silica nanoparticles have been detected in relation to the presence of serum in culture medium. The adsorption of serum proteins to the silica surface could result in altered compatibility and uptake into the cells [28, 29]. Indeed, the serum-driven agglomeration of primary NPs to larger secondary NPs affects cell viability [30], with important implications for the evaluation of the cytotoxic potential of silica NPs, as well as other nanomaterials in standard cell cultures.

In the present study, we explored the toxicity induced by in vitro incubation of three human cell lines with the commercial AS30 and SM30 Ludox® nanoparticles. These colloidal amorphous silica NPs are widely used in various industrial fields, such as in the production of printer’s inks and paints, in textile industry, and in food industry for the fining of drinks. Two of the three cell lines used in our experiments are epithelial cells originated from lungs, A549 cancer cells, and CCD-34Lu normal fibroblasts, chosen because the entry through the respiratory tract is one of the most frequent routes by which nanomaterials may enter the body. The third cell line, HT-1080, derived from human fibrosarcoma, is also used to test the cytotoxicity of nanomaterials [31–34]. We exposed the cells to different treatment modalities, in order to evaluate the influence of serum and the incubation time on Ludox® NPs cytotoxicity. We compared short-time incubation in serum-free medium and a long-time incubation in medium supplemented with serum on the toxicity induced by Ludox® NPs using different assays. Cell viability testing was carried out with the widely used short-term assay (MTS) and the long-term clonogenic assay to obtain a more accurate estimation of the potential toxicity of Ludox® NPs. Our results demonstrate that the choice of the experimental conditions and the toxicity testing protocols plays a relevant role in determining the safe concentrations of potential hazards of nanomaterials.

## Materials and methods

### Materials

Ludox® nanoparticles were obtained from Sigma-Aldrich (Milan, Italy). Ludox® is a registered trademark of W.R. Grace & Co.-Conn. Fetal calf serum (FCS) and 0.05 % trypsin–0.53 mM EDTA were purchased from Gibco (Invitrogen, Italy). F-12 K medium, Dulbecco’s modified Eagle’s medium (DMEM, 0.2 M GlutaMAX, 4.5 g/L glucose) and minimum essential medium (MEM, Earl's salt, l-glutamine) were provided by Gibco (Invitrogen). Penicillin–streptomycin, formaldehyde, RNase, sodium pyruvate, TPEN (*N*,*N*,*N*',*N*'-tetrakis-(2-pyridylmethyl)-ethylenediamine), HEPES (4-(2-hydroxyethyl)-1-piperazineethanesulfonic acid), Triton X-100, and propidium iodide were obtained from Sigma-Aldrich. Goat serum, Hank's balanced salt solution (HBSS), non-essential amino acids (NEAA) 100×, mounting medium Vectashield, carboxy-H_2_DCFDA (carboxy-2′,7′-dichlorofluorescein diacetate) probe were purchased from Invitrogen. DAPI (4',6-diamidino-2-phenylindole) was obtained from Roche Diagnostics (Indianapolis, IN, USA). Anti-γ-H2AX mouse monoclonal antibody was purchased from Upstate, Millipore (Billerica, MA, USA) and from Molecular Probes (Alexa Fluor, Life Technologies, Carlsbad, CA, USA). CellTiter 96®AQueous One Solution Proliferation Assay kit for MTS assay was purchased from Promega (Milan, Italy); ApoAlertCaspase Fluorescent Assay kit was provided by Clontech (Milan, Italy). Annexin-V-FLUOS Staining Kit was bought from Roche Applied Science (Indianapolis, IN, USA). Solvents and commercially available reagents were used as received. Ultrapure deionized water (*R* > 18 MΩ) was prepared using a Milli-Q system (Millipore).

### Ludox® nanoparticles

Ludox® silica nanoparticles of two different sizes, AS30 (ammonium counterion) and SM30 (sodium counterion), were obtained by the commercial source as 30 wt.% suspension in H_2_O. The nanoparticle suspensions were diluted with ultrapure water (Milli-Q) to the desired concentration (30–40 mg/mL), extensively dialyzed into a 75-mL Amicon ultrafiltration cell, equipped with a 10-kDa regenerated cellulose membrane, and finally filtered with 0.22 μm Durapore membrane. Nanoparticle concentration in the purified sample was determined by weighing a dried aliquot of the solution.

Transmission electron microscopy (TEM) images of the particles were obtained with a FeiTecnai 12 transmission electron microscope operating at 100 keV. Samples for TEM were prepared by spreading a droplet of the nanoparticle solution in water (∼1 mg/mL) onto standard carbon-coated copper grids (200-mesh). Dimensional analysis of nanoparticles from TEM images was performed by using the Image J software. No differences were found when nanoparticles for TEM analysis were diluted with water or phosphate-buffered saline (PBS) solution.

Dynamic light scattering (DLS) measurements were performed with a Zetasizer NanoS (Malvern) equipped with a thermostatic cell holder and Ar laser operating at 633 nm. Hydrodynamic particle diameters were obtained from cumulant fit of the autocorrelation functions at 178° scattering angle. Size measurements were performed at 37 °C. DLS measurements where performed only in PBS and in cell culture medium, with or without 3 % of FCS, because the electric double layer produced by the highly negative surface charge of the nanoparticles hampers reliable measurements in pure water.

For the stability tests, Ludox® NPs AS30 and SM30 were diluted in water and in cell culture medium, with or without 3 % of FCS, to final concentrations of 0.1 and 1 mg/mL. Immediately after dilution (0 h) and after 24 h of incubation at 37 °C, the absorption of the suspensions was recorded in the 200–800 nm range. For DLS analyses, NPs were diluted in PBS or in cell culture medium with or without 3 % of FCS, and three size measurements were performed for each sample after 2 h incubation at 37 °C. For cytotoxicity tests, the dialyzed NP stock suspensions were diluted with ultrapure water (5 mg/mL); the pH was adjusted between 7.3 and 7.5 with 1 M HCl, and the suspensions were sterilized by filtration with 0.22 μm (control experiment confirm that such operations do not alter the nanoparticles concentration). The diluted solutions were prepared immediately before use.

### Cell lines

The human cell lines A549 (lung adenocarcinoma), CCD-34Lu (normal lung fibroblasts), and HT-1080 (fibrosarcoma) were obtained from American Type Culture Collection (ATCC, Rockville, USA) and cultured in monolayer. A549, CCD-34Lu, and HT-1080 cells were maintained respectively in F12-K medium, DMEM supplemented with 0.1 mM NEAA, and 20 mM HEPES, and MEM medium supplemented with 0.1 mM NEAA and 1 mM sodium pyruvate. All culture media were supplemented with 10 % heat-inactivated FCS, 38 units/ml streptomycin, and 100 units/ml penicillin G in standard culture conditions and during the post-treatment recovery (complete medium). Cells were kept at 37 °C in a humidified atmosphere containing 5 % CO_2_.

### NPs treatments

To evaluate the cytotoxicity induced by Ludox® NPs, the cells were plated and allowed to attach for 24 h. Then, NPs were diluted to appropriate concentrations and immediately applied to the cells. We used two modalities of treatment: long incubation for 24, 48, or 72 h in culture medium supplemented with 3 % FCS, or short incubation for 2 h in serum-free medium, followed by a post-treatment recovery of 3 or 22 h in complete medium (10 % FCS). NP concentrations (0.005–0.6 mg/mL) were chosen to evaluate the dose/survival according to the treatment conditions. Control cells underwent the same steps of treated cells except for NP exposure.

### Assessment of cytotoxicity

Cytotoxicity induced by Ludox® NPs was evaluated by the MTS assay which measures the reduction of tetrazolium salts to water-soluble formazan product. The intracellular reduction of MTS is primarily attributable to mitochondrial dehydrogenases, and therefore this conversion is conveniently used as a measure of cell viability. Briefly, 8 × 10^3^ cells/cm^2^ were seeded in triplicate in 96-well plates (200 μL/well). After 24 h, the culture medium was removed, and the cells were incubated with 150 μL of medium containing different concentrations of AS30 or SM30 NPs. After predetermined incubation time, the medium containing NPs was removed, and the cells were incubated for 60–90 min in the dark with 20 μL of the MTS reagent diluted in 100 μL of serum-free medium. The absorbance of formazan product was recorded at 490 nm with a microplate reader (Spectramax 190, Molecular Device®). Cell viability was determined by comparing the absorbance values of the treated with those of untreated cells that were considered as 100 %. The potential interaction of Ludox NPs with MTS–formazan crystals has been tested to exclude any interference with the dye.

The cytotoxicity of NPs was also assessed by clonogenic assay that measures the ability of single cells to form colonies. Cells (2–4 × 10^4^ cell/cm^2^) were seeded in 6-cm culture dishes and allowed to attach overnight. Cells were subjected to short and long treatments, harvested by trypsinization, and counted by trypan blue dye exclusion. An appropriate number of viable cells (10.2 cell/cm^2^ of cancer cells) was plated in culture dishes. The 3.2 cell/cm^2^ CCD-34Lu cells were seeded together with feeder layer IMR-90 cells (1.9 × 10^3^ cell/cm^2^) in medium supplemented with 15 % FCS. After 7–14 days at 37 °C, the colonies were counted after staining with 0.4 % crystal violet and counted. Only colonies containing more than 50 cells were scored as survivors. Cell survival was calculated as percentage of cloning efficiency (CE) of treated cells over CE of control cells. To compare the results obtained by MTS and clonogenic assays, the cytotoxicity induced by NPs was expressed as half-maximum effective concentration (EC_50_) in milligrams per milliliter [35].

### Apoptosis detection

The induction of apoptosis in cells treated with Ludox® NPs was analyzed by different assays. The Annexin-V-FLUOS Staining Kit detects the early stage of apoptosis and allows quantification and differentiation from necrosis. Annexin-V–fluorescein is a protein with high affinity for phosphatidylserine (PS), while propidium iodide crosses only damaged plasma membrane and intercalates to DNA. Briefly, cells were treated with 0.04 mg/mL SM30 for 2 h in serum-free medium, and after a recovery of 3 and 22 h in complete medium, the cells were detached and centrifuged at 200×*g* for 5 min. The pellet was resuspended in 100 μL of Annexin-V–Fluos labeling solution (20 μL of Annexin-V–Fluos labeling reagent and 20 μL of propidium iodide solution in 1 mL incubation buffer) and incubated for 10 min at 37 °C. Samples were analyzed by flow cytometry with a FACSCanto™ II flow cytometer (BD Bioscences, San Jose, CA, USA).

The formation of apoptotic bodies was investigated by DAPI staining after treatment with both AS30 or SM30 NPs (0.04 mg/mL) for 2 h in serum-free medium followed by a recovery of 22 h in complete medium. After rinsing with HBSS twice, the cells were fixed (9:1 absolute ethanol/acetic acid) on ice and centrifuged. This step was repeated four times. After overnight incubation at 4 °C, cells were stained with 0.2 μg/mL DAPI. At least 1,000 nuclei for each time point were inspected by fluorescence microscopy for detecting the typical morphological appearance of chromatin condensation during the late step of apoptosis with a Leica DM 5000B microscope (Leica Microsystems).

Apoptosis induction was measured also by the caspase-3 activation using the ApoAlert® Caspase Fluorescent Assay kit according to manufacturer’s instructions and as previously described [36]. Cell lysates (1 × 10^6^ cells) were prepared at the end of 2 h treatment in serum-free medium followed by a recovery of 22 h in complete medium and analyzed with a Perkin-Elmer LS-50 B spectrofluorimeter. Cells treated for 5 h with TPEN (*N*,*N*,*N*',*N*'-tetrakis-(2-pyridylmethyl)-ethylenediamine, 30 μM) were used as positive control.

### Reactive oxygen species (ROS) measurements

The production of intracellular reactive oxygen species (ROS) was measured using the probe 6-carboxy-2′,7′-dichlorodihydrofluorescein diacetate (carboxy-H_2_DCFDA). Cells (CCD-34Lu and A549, 1.8 × 10^4^ cell/cm^2^; HT-1080, 7 × 10^3^ cell/cm^2^) were seeded in 35-mm-diameter tissue culture dishes and allowed to attach for 24 h. Thereafter, the medium was replaced with fresh serum-free medium containing Ludox® AS30 or SM30 NPs (0.02–0.06 mg/mL). After 2 h of treatment, the medium was discarded, and the cells were immediately analyzed for ROS detection or incubated for 3 or 22 h in complete medium before analyses. The cells were washed with PBS and incubated with carboxy-H_2_DCFDA (25 μM) diluted in PBS for 40 min at 37 °C in the dark. The cells were washed, harvested, and then analyzed by a BD FACSCanto II flow cytometer (Becton Dickinson; Biosciences). The fluorescence intensities were measured using a 488 nm laser and fluorescein isothiocyanate (FITC) detection channel (530 ± 15 nm) by acquiring 10.000 events/sample. Cells incubated for 2 h in serum-free medium without NPs were used as negative controls. The mean fluorescence intensity of cells treated with NPs (0.02 to 0.06 mg/mL) was expressed as percentage of controls. Selected samples were also stained with propidium iodide (50 μg/mL, fluorescence detection at 585 ± 21 nm) to evaluate the integrity of the plasma membrane.

### Induction of DNA double-strand breaks

The induction of DNA double-strand breaks (DSBs) by incubation with NPs was assessed by the presence of γ-H2AX foci over the nucleus. Cells (7 × 10^3^ cell/cm^2^ HT-1080, 1.2 × 10^4^ cell/cm^2^ CCD-34Lu, and A549 cells) were seeded in 35-mm-diameter tissue culture dishes containing a glass coverslip and allowed to attach for 24 h. Thereafter, the cells were treated with AS30 and SM30 NPs (0.01–0.4 mg/mL) in medium with 3 % serum (24, 48, and 72 h), or in serum-free medium for 2 h, and fixed immediately at the end of treatments or maintained for 3 or 22 h in NP-free complete medium. Cells were rinsed twice in PBS and fixed in formaldehyde 4 % in PBS 1× at 37 °C for 15 min. After washing in PBS, the cells were permeabilized in 0.2 % Triton X-100 for 10 min at 37 °C and incubated in 10 % goat serum in PBS for 90 min at room temperature to suppress non-specific antibody binding. The cells were then incubated for 90 min at room temperature in 30 μL volume of 10 % goat serum containing 1:200 dilution of phospho-specific (Ser-139) histone H2AX (γ-H2AX) mouse monoclonal antibody. The slides were washed twice with PBS and then incubated in 30 μL of 10 % goat serum containing 1:250 dilution of Alexa Fluor 488 goat anti-mouse, for 1 h in the dark. After washing in PBS, the dry samples were mounted with mounting medium Vectashield, counterstained with DAPI 0.2 μg/mL, and analyzed by fluorescence microscopy with Leica DM 5000B microscope. At least 100 cells were scored for each time point, and cells with more than four foci per nucleus were considered positive.

## Results

### Characterization of Ludox® AS30 and SM30

AS30 and SM30 commercial Ludox® nanoparticles were selected for two reasons: (1) They have different sizes (see infra), and (2) they are stabilized by different counterions, namely ammonium for AS30 and sodium for SM30. Such differences should allow a better discrimination between toxicity arising from the silica nanoparticles and from possible contaminants. In addition, samples from commercial source were submitted to extensive dialysis to remove any possible contaminant. DLS and TEM analyses, performed before and after the dialysis, confirmed that the purification procedure does not alter the size and morphology of the nanoparticles. The hydrodynamic diameters, obtained by DLS, were 20 ± 4 and 14 ± 4 nm for Ludox® AS30 and SM30, respectively. The mean nanoparticle sizes determined by TEM micrographs were 18 ± 3 (AS30) and 9 ± 3 nm (SM30). Zeta potential of both NPs was negatively charged, −25.9 mV and −26.3 for Ludox® AS30 and SM30, respectively, indicating that the two preparations of Ludox® NPs have a similar stability. The data relative to Ludox® nanoparticles characterization are available (see Electronic supplementary material Fig. S1).

The behavior of nanoparticles in different media was preliminarily investigated by incubating NPs in pure water, in culture medium, and in culture medium supplemented with low concentration (3 %) of serum. Spectra recorded by UV/vis spectroscopy in water and culture media do not show any detectable absorbance even after 24 h, as expected on the basis of the silica properties and the small size of the nanoparticles. When serum is present, an unstructured absorbance typical of scattering is immediately observed, and its intensity increases after 24 h (data not shown). Such a behavior is likely an indication of the formation of nanoparticle aggregates driven by the presence of serum proteins.

**Fig. 1:**
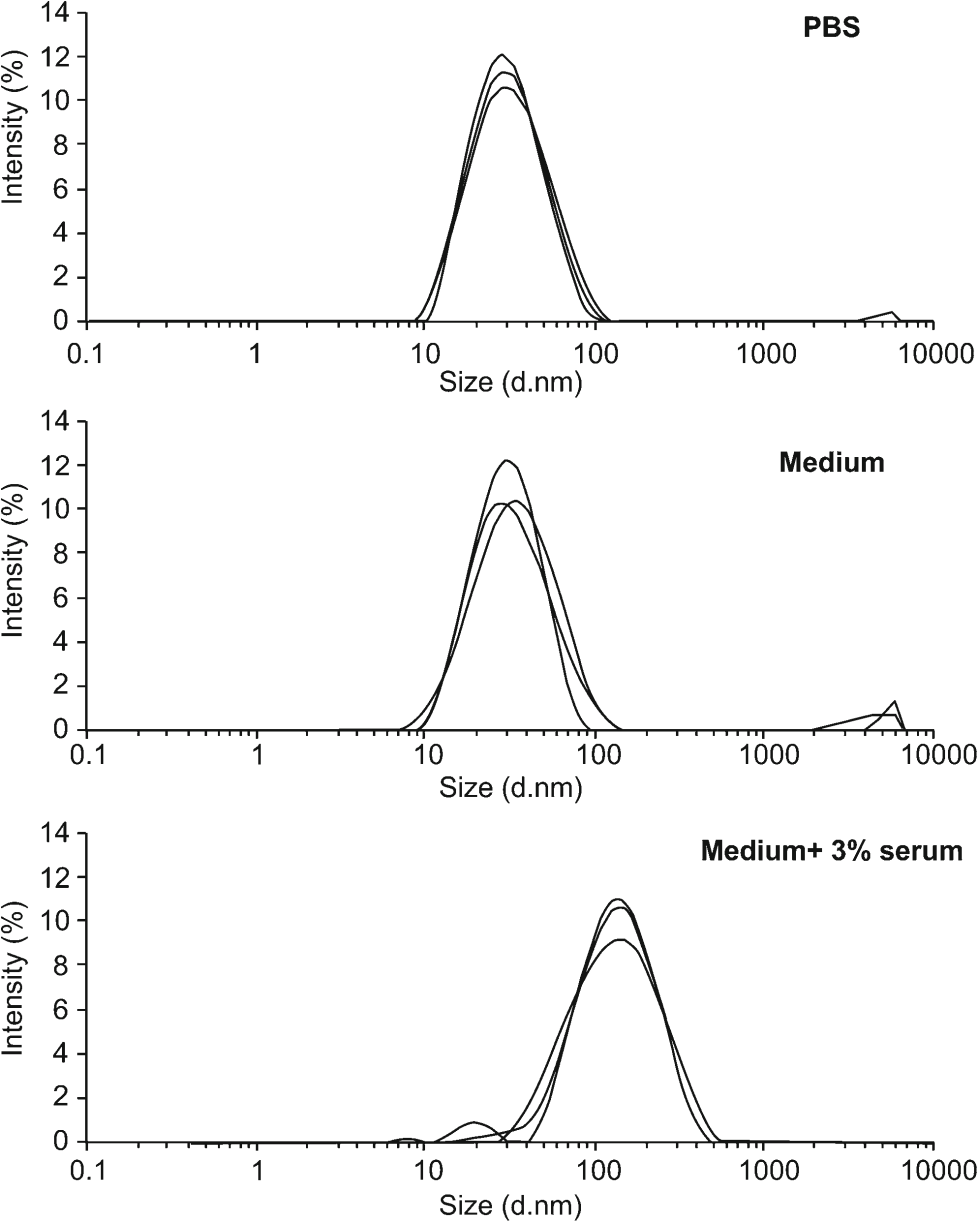
Dynamic light scattering (DLS) particle size distribution of Ludox® NPs SM30 (1 mg/mL) suspended in PBS, in culture medium, and in medium with 3 % of serum. The measures were performed for each sample after 2 h incubation at 37 °C

This hypothesis was confirmed by measuring the NP sizes with DLS upon incubation in different media (Fig. 1). Again, the intensity-weighted distribution curves of SM30 NPs, at concentration of 1 mg/mL, in PBS solution and in culture medium without serum were very similar to each other and at any time interval, showing an average diameter of about 20 nm, a value larger than 14 nm reported in Fig. S1, since intensity-weighted distribution plots usually slightly overestimate sizes. After addition of low concentration of serum (3 %) to SM30 suspension in culture medium, larger objects were detected by the DLS analysis, with an average size of 110 nm and large size dispersion. Such a behavior can be likely ascribed to the formation of nanoparticle aggregates with serum components. Similar results were obtained for suspensions at lower concentrations of SM30 NPs (0.1 mg/mL) and for Ludox® AS30 (data not shown).

### Cytotoxicity of Ludox® nanoparticles

Cultures of CCD-34Lu, HT-1080, and A549 cells were incubated with increasing concentrations of Ludox® NPs (AS30 and SM30) by adopting two treatment modalities: incubation for long times (24, 48, and 72 h) in medium supplemented with 3 % of serum, or incubation for short time (2 h) in serum-free medium. We selected these treatment modalities because DLS measurements showed that NPs aggregate in presence of serum (Fig. 1), and preliminary cell viability tests suggested that 2 h is the maximum time interval of culture in medium, without serum, tolerated by the most sensitive cell line (CCD-34Lu) here analyzed (data not shown). For long incubation times, we supplemented culture medium with 3 % of serum, which represents the lower percentage suitable for maintaining the cells up to 72 h without suffering, in accordance with our previous observations [37].

**Fig. 2:**
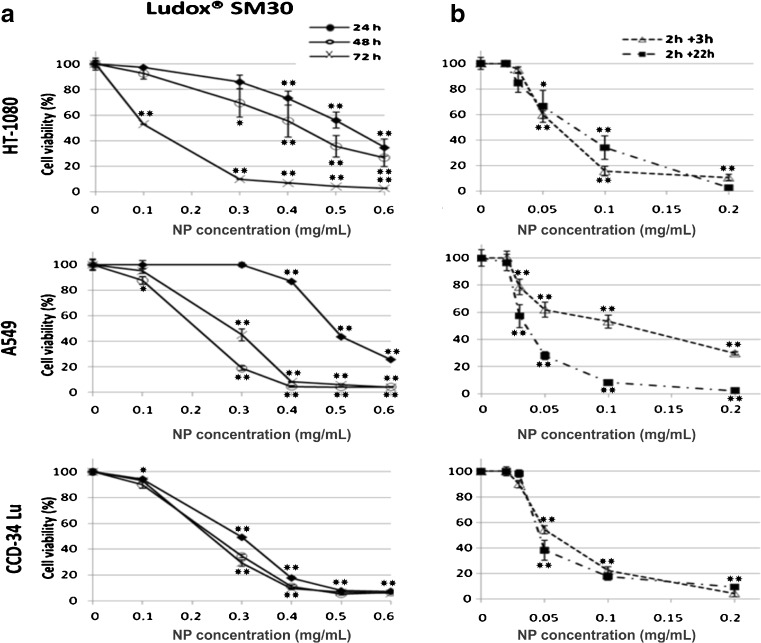
Cell viability measured by MTS assay in HT-1080, A549, and CCD-34 Lu cells treated with increasing concentrations of Ludox® NPs SM30 in medium with 3 % of serum (**a**) or without serum, followed by a recovery for 3 or 22 h in complete medium (10 % of serum) (**b**). The data represent mean ± SD (3 ≤ *n* ≤ 15). *p < 0.05, **p < 0.01 (*t* test; treated vs. control cells)

**Fig. 3:**
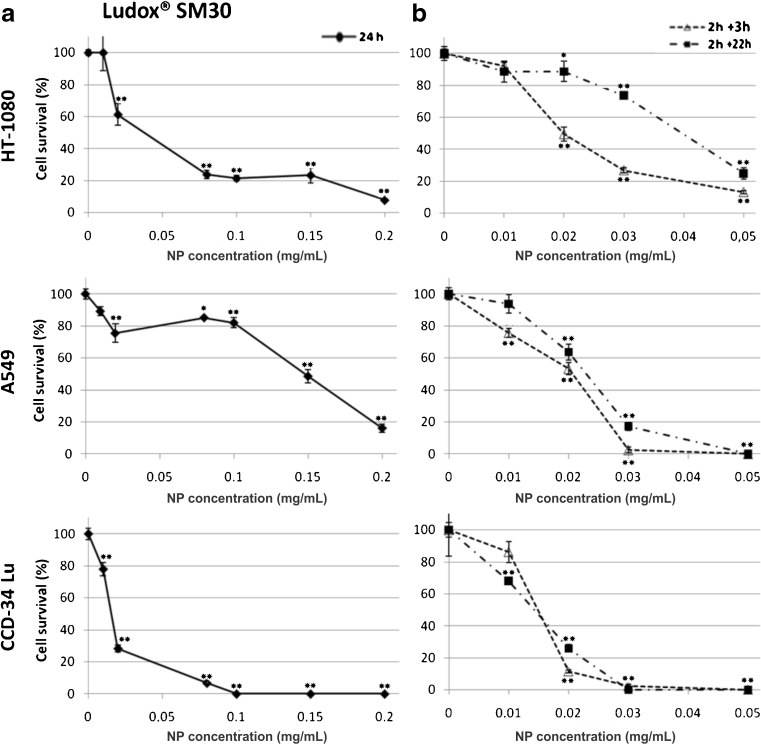
Cell survival measured by clonogenic assay in HT-1080, A549, and CCD-34Lu cells treated with increasing concentrations of Ludox® NPs SM30. Cell cloning was performed after a 24-h treatment with NPs in medium containing 3 % of serum (**a**), or after a 2 h treatment in serum-free medium followed by a recovery for 3 or 22 h in complete medium (**b**). The data represent mean ± SD (3 ≤ *n* ≤ 12). *p < 0.05, **p < 0.01 (*t* test; treated vs. control cells)

**Fig. 4:**
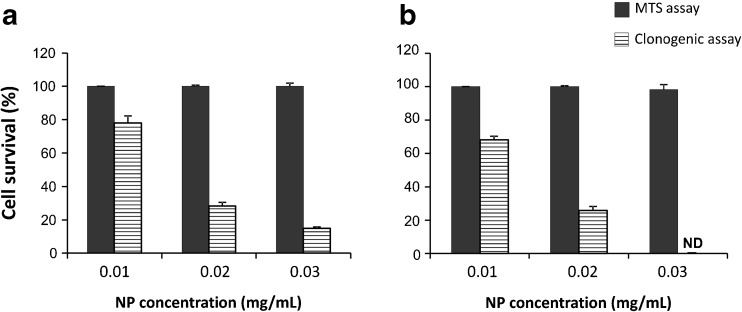
Cell survival of CCD-34 Lu assessed by MTS and clonogenic assays. The cells were incubated with Ludox® SM30 for 24 h in medium with 3 % of serum (**a**) or for 2 h in serum-free medium followed by a recovery of 22 h in complete medium (**b**). The data represent mean ± SD from four independent experiments. Cell survival determined by clonogenic assay was significantly lower than that determined by MTS for all the three tested doses (*p* < 0.001, *t* test, clonogenic vs MTS). *ND* = not detectable

**Fig. 5:**
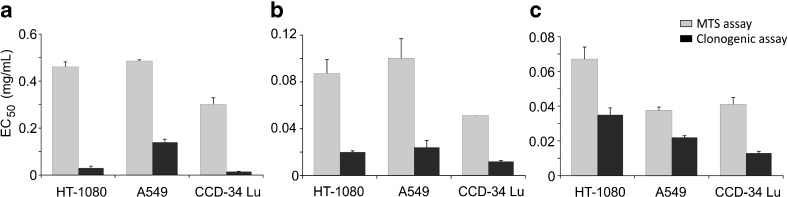
Cytotoxicity of SM30 NPs expressed as half-maximum effective concentration (EC_50_ value in milligrams per milliliter), as assessed by MTS and clonogenic assays. **a** Treatment of 24 h in medium with 3 % of serum; **b** treatment of 2 h in serum-free medium, followed by recovery of 3 h in complete medium (serum 10 %) or of 22 h in complete medium (**c**). The data represent mean values of EC_50_ *±* SD (3 ≤ *n* ≤ 15). In all treatment conditions and in all cell lines, the values of EC_50_ derived from clonogenic assay were significantly lower than those determined by MTS (*p* < 0.001, *t* test)

The results of MTS assay showed that the exposure to NPs caused a significant decrease of cell viability in a dose- and time-dependent manner, and the incubations in serum-free medium were the most toxic (Fig. 2). Under this treatment condition, cell viability strongly decreased at NP concentrations at which the majority of cells survived when the treatment occurred in presence of serum. For example, the cell viability of CCD-34Lu was about 90 % after incubation with 0.1 mg/mL of SM30 in medium supplemented with serum and only 20 % in medium without serum. The colony-forming ability of cells treated with nanoparticles has been assessed after treatments with both modalities (Fig. 3). The results confirmed that the absence of serum during the treatment increased the toxicity of silica nanoparticles. With few exceptions, in our experiments, SM30 and AS30 NPs caused very similar levels of cytotoxicity. Thus, we reported here only the results obtained with Ludox® SM30. The data of cell viability after treatments with Ludox® AS30 are available (see Electronic supplementary material Fig. S2 and S3). The viability of CCD-34Lu cells analyzed by MTS seems to be substantially unaffected by treatment with 0.01–0.03 mg/mL of SM30. In contrast, the results of clonogenic assay performed under the same conditions markedly reduced cloning efficiency (Fig. 4). To compare the results obtained from the two assays, we calculated the concentrations of SM30 NPs able to reduce cell viability to 50 % of the control cells (EC_50_ value). As expected, with both assays, the toxicity induced by NP incubation in medium without serum resulted in EC_50_ values lower in comparison with treatments carried out in the presence of serum (Fig. 5). Moreover, in all treatment conditions and in all cell lines, the clonogenic assay was more sensitive than the MTS assay, as shown by the EC_50_ values significantly lower.

### Oxidative stress induced by Ludox® NPs

**Fig. 6:**
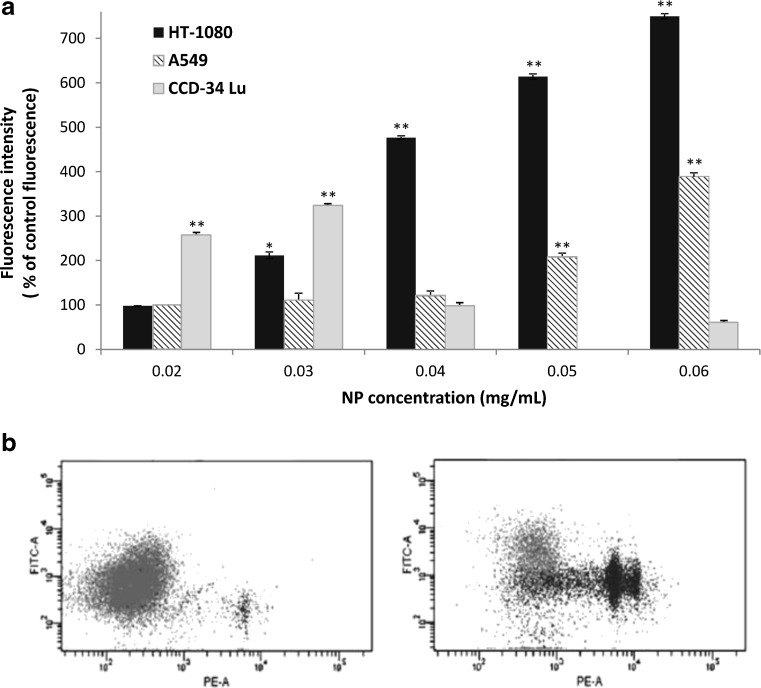
Reactive oxygen species (ROS) generation in cells treated with Ludox SM30 for 2 h in serum-free medium. **a** The mean florescence intensity of the ROS probe (carboxy-DCF) is expressed as percentage of control fluorescence. The data represent mean ± SE (*n* = 3). *p < 0.05, **p < 0.01 (*t* test; treated vs. control cells). **b** *Dot plots* obtained from representative experiment, showing propidium iodide (PE) versus carboxy-DCF fluorescence (FITC) in CCD-34Lu cells; *left panel*: untreated cells; *right panel*: cells treated with SM30 (0.03 mg/mL) for 2 h in serum-free medium

The formation of intracellular ROS induced by NP treatment was evaluated by measuring the fluorescence intensity emitted by 2',7'-dichlorofluorescein (DCF) formed from the interaction of H_2_DCFDA with ROS. The level of ROS was measured after both treatment modalities (not shown), but a significant increase of DCF florescence has been detected only when the measurements were performed immediately at the end of 2-h treatment in serum-free medium (Fig. 6a). The mean fluorescence intensity (MFI) of cells treated with NPs (0.02 to 0.06 mg/mL) significantly increased in the two cancer cell lines, A549 and HT-1080. At the highest concentration of SM30 (0.06 mg/mL), the MFI was about seven- and fourfold over the control respectively in HT-1080 and in A549 cells. In CCD-34Lu cells, MFI significantly increased over the control at very low NP concentration (0.02 mg/mL), reached the maximum value at 0.03 mg/mL, and markedly decreased at higher concentrations. To evaluate whether the decrease of the ROS level in CCD-34Lu was correlated to a decrease of cell viability, we measured the plasma membrane permeability to propidium iodide (PI) added during the incubation with the ROS probe. The dot plots of PI fluorescence versus DCF fluorescence (Fig. 6b) shows that about 33 % of cells exposed to 0.03 mg/mL of SM30 were positive to PI fluorescence, because of the loss of plasma membrane integrity. The cells positive to PI and negative to carboxy–DCF were probably unable to convert the ROS probe to fluorescent compound. In contrast, most HT-1080 and A549 cells, in which ROS level increased with NP concentration, were viable since they were negative to PI (not shown).

### Apoptosis induction by Ludox® NPs

**Fig. 7:**
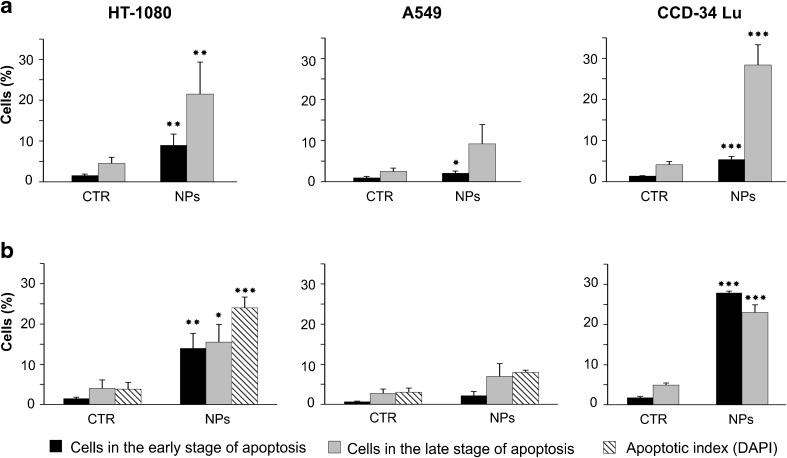
Apoptosis induction in cells treated with Ludox® SM30 (0.04 mg/mL) for 2 h in serum-free medium, followed by a recovery of 3 (**a**) or 22 h (**b**) in complete medium. After the recovery, the cells were double-stained with Annexin V–FITC/propidium iodide and analyzed by flow cytometry to detect cells in the early or in the late stage of apoptosis. Data represent means ± SD (*n* = 3). *p < 0.05, **p < 0.01, ***p < 0.001 (*t* test; treated vs. control cells). The presence of apoptotic bodies (apoptotic index) was checked by DAPI staining at the end of 2 + 22 h treatment (***p < 0.01, *t* test, treated vs. control cells)

We investigated the modality of cell death induced by treatment with Ludox® NPs by the Annexin V–FITC/propidium iodide double staining followed by flow cytometry analysis. Upon activation of the apoptotic program, cells lose the asymmetry of the plasma membrane, by translocating the phospholipid PS on the outer leaflet of the membrane. The double staining with Annexin V and propidium iodide allows to distinguish cells undergoing early apoptosis (positive to only Annexin V–FITC) and cells in late stage of apoptosis (positive to Annexin V–FITC/propidium iodide) from necrotic cells (positive to only propidium iodide). The analyses were performed in cells exposed for 2 h to SM30 (0.04 mg/mL) suspended in serum-free medium (Fig. 7) since, under this treatment condition, the loss of cell viability and the formation of intracellular ROS were much more pronounced. The fraction of CCD-34Lu cells positive to only Annexin V increased during post-treatment incubation from 34 % (3 h) to 51 % (22 h), considering the total cells in early and late stage of apoptosis. In cancer cells, this fraction was lower at both time points after treatment: 30 % in HT-1080 cells and 9–11 % in A549 cells. We also checked by DAPI staining the cells treated for 2 h with SM30 NPs followed by 22 h of post-treatment incubation for presence of apoptotic bodies formed during the late phase of apoptosis (Fig. 7a). Apoptotic index, calculated as percentage of apoptotic bodies, significantly increased in HT-1080 and in A549 cells but not in CCD-34Lu (Fig. 7b); moreover, no apoptotic bodies were detected when the three cell lines were subjected to long NP-incubation in presence of serum 3 % (not shown).

The induction of apoptosis in HT-1080 cells was caspase-dependent, as detected by fluorimetric assay of caspase-3 activation performed at the same time of DAPI staining (2 h + 22 h). In this cell line, the activation of caspase-3 increased eight times over control cells whereas, in A549 and CCD-34Lu, the fluorescence intensity was almost the same as in control (data not shown).

### DNA double-strand breaks induced by Ludox® nanoparticles

**Fig. 8:**
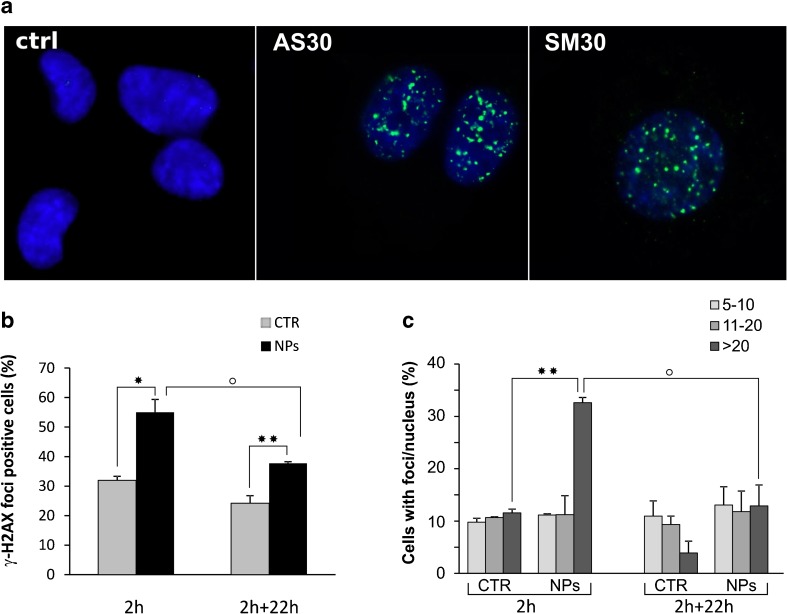
Induction of DNA double-strand breaks in HT-1080 cells. **a** Immunofluorescence of γ-H2AX foci in HT-1080 treated with Ludox ® AS30 and SM30 NPs in serum-free medium. **b** Percentage of HT-1080 cells positive for γ-H2AX foci after 2 h incubation with 0.04 mg/mL of SM30 NPs in serum-free medium. Cells were fixed at the end of treatment (2 h) and after a recovery of 22 h in complete medium (2 + 22 h). **c** Positive cells for γ-H2AX foci were categorized on the basis of the number of foci/nucleus (5–10, 11–20, >20 foci). Data represent means ± SD (2 ≤ *n* ≤ 4). *p < 0.05, **p < 0.01, *t* test, treated versus control cells; **°** *p* < 0.05, *t* test, (2 h) versus (2 + 22 h) treated cells

The DNA-damaging effects of NPs were assessed on the basis of the induction of DNA DSBs by scoring nuclei for the presence of foci of histone γ-H2AX, a reliable marker of DSBs. The cells were incubated with SM30 and AS30 NPs (0.01–0.4 mg/mL) in medium serum-free or supplemented with serum. No foci were detected in CCD-34Lu and A549 cells under all treatment conditions, as well as in HT-1080 cells incubated with NPs in medium supplemented with serum (data not shown). On the contrary, a consistent number of γ-H2AX foci was detected when HT-1080 cells were exposed to NPs in serum-free medium (Fig. 8a). In Fig. 8b, we reported the percentage of foci-positive cells at the end of 2 h incubation with 0.04 mg/mL of SM30 and after 22 h of recovery in complete medium. The fraction of cells positive for γ-H2AX foci grew from 32 % in untreated cells to 55 % in treated cells at the end of 2 h incubation and significantly decreased 22 h after (38 %). The rejoining of DNA double-strand breaks was also analyzed on the basis of the number of foci/nucleus. HT-1080 cells positive for γ-H2AX foci were classified in three groups having 5–10, 11–20, and more than 20 foci/nucleus. Figure 8c shows that the cells with more than 20 foci/nucleus were 33 % at the end of treatment and decreased to 13 % 22 h later, fitting the progression of DNA repair.

## Discussion

Although nanomaterials are applied in many fields that seem to be destined to increase, the mechanisms involved in the induction of cytotoxicity remain not completely clarified. The purpose of our work was to evaluate the level of the in vitro cytotoxicity induced by commercial silica nanoparticles of two different sizes, Ludox® SM30 and AS30. We used DLS and TEM to evaluate size distribution, state of dispersion, and Zeta potential of Ludox® NPs prior to setting up the experiments with three different cell lines. The little differences in particle sizes measured by DLS (AS30, 20 ± 4 nm; SM30, 14 ± 4 nm) and TEM (AS30, 18 ± 3 nm; SM30, 9 ± 3 nm) reflect the typical difference between the mean hydrodynamic diameter (measured by DLS) and the “real” size (measured by TEM), the first being larger, as usually reported for particles in solution [38]. The Zeta potential values are above the −30 mV threshold commonly considered to ensure stability to a dispersion of nanoparticles stabilized by electrostatic repulsion forces. Still, in preliminary experiments, we did not detect any aggregation either in PBS or in culture medium. On the other hand, Ludox® NPs strongly aggregated when the medium was supplemented with serum, even in small amounts (3 %), and even with very low NP concentrations (0.01 mg/mL, not shown). Such a behavior is completely consistent with the well-known protein flocculation ability of silica nanoparticles that is exploited in many applications as beverage clarification. The interaction of NPs with serum proteins results in formation of large aggregates with an average size of 110 nm, as resulting from the DLS analysis reported in Fig. 1, immediately after diluting NPs with medium supplemented with 3 % of serum. Likely, the aggregation process continues with the time of incubation, as suggested by the increased scattering observed in NP suspensions by UV/visible absorption experiments (data not shown). The adsorption of plasma proteins onto the surface of nanostructures represents a well-known problem for the successful application of nanobiotechnology and nanomedicine [39], and many studies have been performed during the last few decades on passivating surfaces of nanomaterials [40–42].

In order to assess the cytotoxicity of Ludox® NPs, we exposed cells to different incubation strategies: short incubation (2 h) in serum-free medium or long incubation (24–72 h) in serum-containing medium. This choice is related to the importance of considering either the time of incubation with NPs, and the presence/absence of serum during treatments, as significant variables in assessing NPs toxicity, in accordance with literature data [30, 43]. Indeed, when the nanoparticles enter the body, the cell–nanoparticles interactions occur through biological protein-rich fluids, as well as in protein-free or protein-poor conditions. The duration of incubation time in serum-free medium and the percentage of serum (3 %) supplemented in the long incubation protocols were checked in our preliminary experiments to assure that such conditions did not affect by themselves cell viability (not shown). As expected, cell treatments performed with Ludox® NPs suspended in medium with or without serum gave different results. Cell viability assays showed little or lower cytotoxicity when treatments occurred in presence of serum, suggesting that NP aggregation induced by serum components decreased their toxicity. Our results are in accordance with those reported in 3T3 cells treated with silica NPs in presence of increasing concentrations of serum [30], probably as a consequence of the lower cellular uptake of NPs suspended in serum-containing medium compared with serum-free medium [29]. A lower level of cytotoxicity has been observed in a murine macrophage cell-line exposed to manufactured NPs (polystyrene beads) suspended in medium-containing serum than in medium without serum [43]. We believe that, when NPs are monodisperse or form small aggregates, they penetrate across cell membrane, and the deleterious effects are caused by the accumulation of NPs in the cytoplasm or in vesicles, as observed for other silica nanoparticles with similar sizes [30, 35, 44]. Under long treatment modality, NPs form aggregates that probably sediment over cell monolayers, without penetrating into the cells. Therefore, the cytotoxicity observed following long NP incubations is very likely caused by damages on plasma membrane that impair its functions. The variation of cytotoxicity of silica NPs as a function of their agglomeration behavior has been reported also in HeLa cells [45] and in blood cells [46].

Previous reports [10, 12, 14] have shown that NPs with small diameter and large surface area/volume ratio induce higher cytotoxicity in comparison with the larger NPs, probably because they were easily internalized by the cells, and, at the same weight/volume of the medium, they were also administered in larger number. The dimensions of Ludox® NPs used in the present work are quite similar, being SM30 9 nm, in accordance with previous results [35], and AS30 18 nm. In our experiments, the different stabilizing counterions did not affect the toxicity induced by NPs; indeed, with few exceptions, SM30 and AS30 NPs caused very similar levels of cytotoxicity, in accordance with their similar sizes (Fig. S1).

To determine the critical concentrations for the exposures to nanomaterials, a careful selection of testing strategies is also required. The most common methods used in assessing the in vitro cytotoxicity of nanomaterials are colorimetric assays (i.e., MTT, MTS, XTT, etc.), in which tetrazolium salts are reduced to formazan by metabolically active cells, producing measurable color changes proportional to the number of viable cells. Although useful to assess cell viability, these assays provide little input in determining the retention of proliferation ability of treated cells. Indeed, they measure cell viability as a function of metabolic activity of cellular dehydrogenases, without considering cell cycle perturbations and cell proliferation alterations. As a consequence, the cytotoxic potency of nanoparticles could be underestimated by the results from short-term assays. For this reason, we assessed the cytotoxicity of Ludox® NPs also with the long-term clonogenic assay, based on the number of colonies formed from single cells. By comparing the results obtained by the two assays, we observed that EC_50_ calculated from clonogenic assay was always lower than that measured by MTS assay. In particular, in HT-1080 and CCD-34Lu cells treated with long treatment modality, the values of EC_50_ were 20- to 30-fold lower when calculated from the data of clonogenic than MTS assay, and two- to fivefold lower in all the three cell lines subjected to short treatment modality (Fig. 5). This result reflects the different sensitivity of MTS and clonogenic assays, based the first on enzymatic activities detected either in viable and in senescent/dying cells, and the second on the retention, by only viable and healthy cells, of proliferation ability. Moreover, by performing clonogenic assays at 3 or 22 h from the end of NP incubation, we obtained information on cellular recover from stress induced by treatments (Fig. 3b). The survival of CCD-34Lu and A549 cells was very similar at both time points, suggesting that these cell lines did not recover during post-treatment incubation and the toxicity induced by NP treatment persisted for long time. Instead, HT-1080 cells recovered part of their proliferation ability during the post-treatment incubation, at least when the NP concentration was low (<0.03 mg/mL). When cells were incubated for 24 h in medium supplemented with 3 % of serum, cell survival similarly decreased with dose in HT-1080 and CCD-34Lu cells, and at the dose 0.1 mg/mL, only 0–20 % survived, in contrast to 80 % of A549 cells (Fig. 3a). Noteworthy are our results showing that NP concentrations, which seem non-toxic on the basis of MTS data, are instead able to inhibit cell proliferation at doses threefold lower.

The toxicological mechanisms of Ludox® NPs were different among the three cells lines assayed in our experiments. Many studies reported the oxidative stress as the main mechanism of silica nanoparticle-induced toxicity responsible for cell damages [12, 15, 16, 18, 22, 44, 47]. In CCD-34Lu, intracellular ROS generated by NP treatments were detectable only at low concentrations (up to 0.03 mg/mL), while, at higher doses, ROS production increased weakly over control (Fig. 6). It seems likely that, in these cells, the mortality induced by NPs was due to the high sensitivity of their plasma membrane, which became severely damaged probably as a consequence of lipid peroxidation, as observed in other cell line exposed to different kind of nanoparticles [48, 49]. Indeed, in treated CCD-34Lu, we observed that phosphaditylserine translocated to the outer leaflet of plasma membrane, but the progression of apoptotic program was halted by the loss of plasma membrane integrity, demonstrated by the propidium iodide staining. As a consequence, apoptotic bodies, which represent the final step of apoptosis, were missing in these cells that probably switched to necrosis.

In cancer cells, and in particular in HT-1080, ROS production was higher than in normal fibroblasts and increased with NP concentration at least up to the highest tested dose. It was observed that intracellular ROS can cause DNA damages, in the form of single- and double-stranded DNA breaks, base modifications, and DNA cross-links, all of which are involved in initiating and promoting carcinogenesis [50]. Moreover, high ROS concentrations are able to activate caspase-3 [49, 51, 52], the pivotal protein in the last phase of apoptosis. At the end of incubation with 0.04 mg/mL of Ludox® SM30 in medium without serum, ROS level in HT-1080 cells significantly increased over control, as well as caspase-3 activity, apoptotic index, and DNA double-strand breaks. A549 cells subjected to the same treatment conditions showed moderate increases of intracellular ROS and apoptosis, and no induction of DNA strand breaks, in accordance with data from the same cells subjected to long incubation with high concentrations of multi-walled carbon nanotubes or silica nanoparticles [38, 53, 54]. The induction of oxidative stress responses have been reported also in a neuronal cell line after exposure to Ludox AS-20 and AM nanoparticles [55].

On the whole, our data show that Ludox® NPs suspended in medium supplemented with serum are unstable and tend to form aggregates, which are toxic for all the three cell lines at concentrations five to tenfold higher than when administered as monodisperse suspensions in serum-free medium. Notably, under short and long treatment modalities, NP concentrations which seem non-toxic on the basis of MTS data are instead able to inhibit cell proliferation at doses at least threefold lower. Our findings are particularly valid for proliferating cells of regenerating epithelia of respiratory and gastrointestinal tracts, where the exposure to nanoparticles can occur by inhalation and ingestion. Indeed, inhaled or ingested NPs may translocate toward the inner tissues, inducing toxicity to proliferating and stem cells of such tissues.

In conclusion, our results highlight the importance of the choice of the testing assays when evaluating cytotoxicity of silica NPs in cell cultures. Indeed, we provide evidence that long-term cytotoxicity assays represent a more appropriate method for accurate and efficient testing of the potential hazards of nanomaterials. Therefore, proper studies comparing the toxicity data obtained with both short-term and long-term assays should be employed when measuring the cell response to nanoparticle exposure.

## Electronic supplementary material

Below is the link to the electronic supplementary material.

MOESM (PDF 9721 kb)
