# Supplementary material for: The toxicity outcome of silica nanoparticles (Ludox®) is influenced by testing techniques and treatment modalities
Source: Anal Bioanal Chem. 2012 Jul 22;404(6):1789–802. doi: 10.1007/s00216-012-6246-6 (PMC3462312; doi:10.1007/s00216-012-6246-6)

Analytical and Bioanalytical Chemistry

Electronic Supplementary Material

The toxicity outcome of silica nanoparticles (Ludox®) is influenced by testing techniques and treatment modalities

Caterina Fede, Francesco Selvestrel, Chiara Compagnin, Maddalena Mognato, Fabrizio Mancin, Elena Reddi, Lucia Celotti

Fig.S1. TEM images and characterization data of SM30 and AS30 Ludox® NPs.

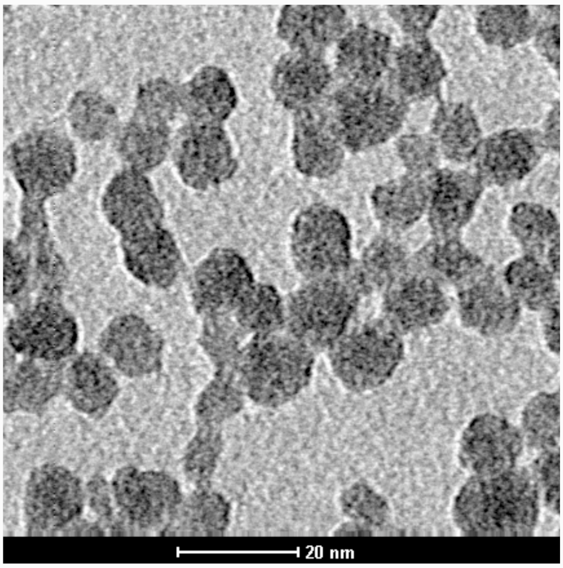

SM30

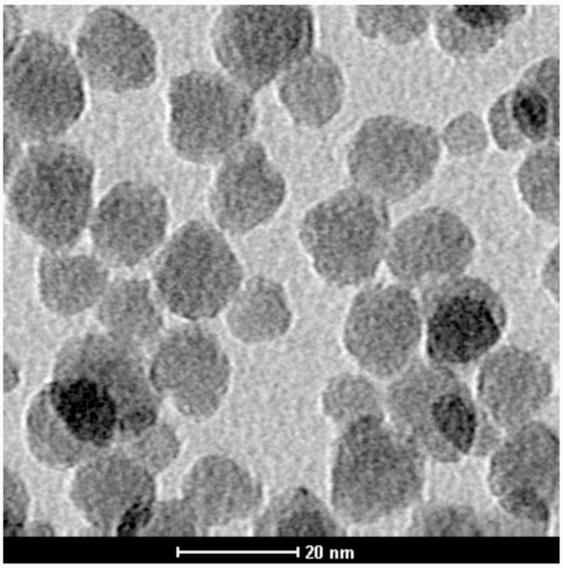

AS30

	Counterion*	ζ potential in PBS	DLS diameter in PBS	Diameter from TEM in PBS	Surface area*	pH*
**SM30**	sodium	-26.3 mV	14 ± 4 nm	9 ± 3 nm	345 m^2/g	10.0
**AS30**	ammonium	-25.9 mV	20 ± 4 nm	18 ± 3 nm	230 m^2/g	9.1

* Data provided by Sigma-Aldrich

Fig. S2. Cell viability measured by MTS assay in HT-1080, A549 and CCD-34 Lu cells treated with increasing concentrations of Ludox® NPs AS30 in medium with 3% of serum (a) or without serum, followed by a recovery for 3 or 22h in complete medium (10% of serum) (b). The data represent mean ± S.D. ($3 \leq n \leq 15$). *$p < 0.05$, **$p < 0.01$ (t test; treated vs. control cells).

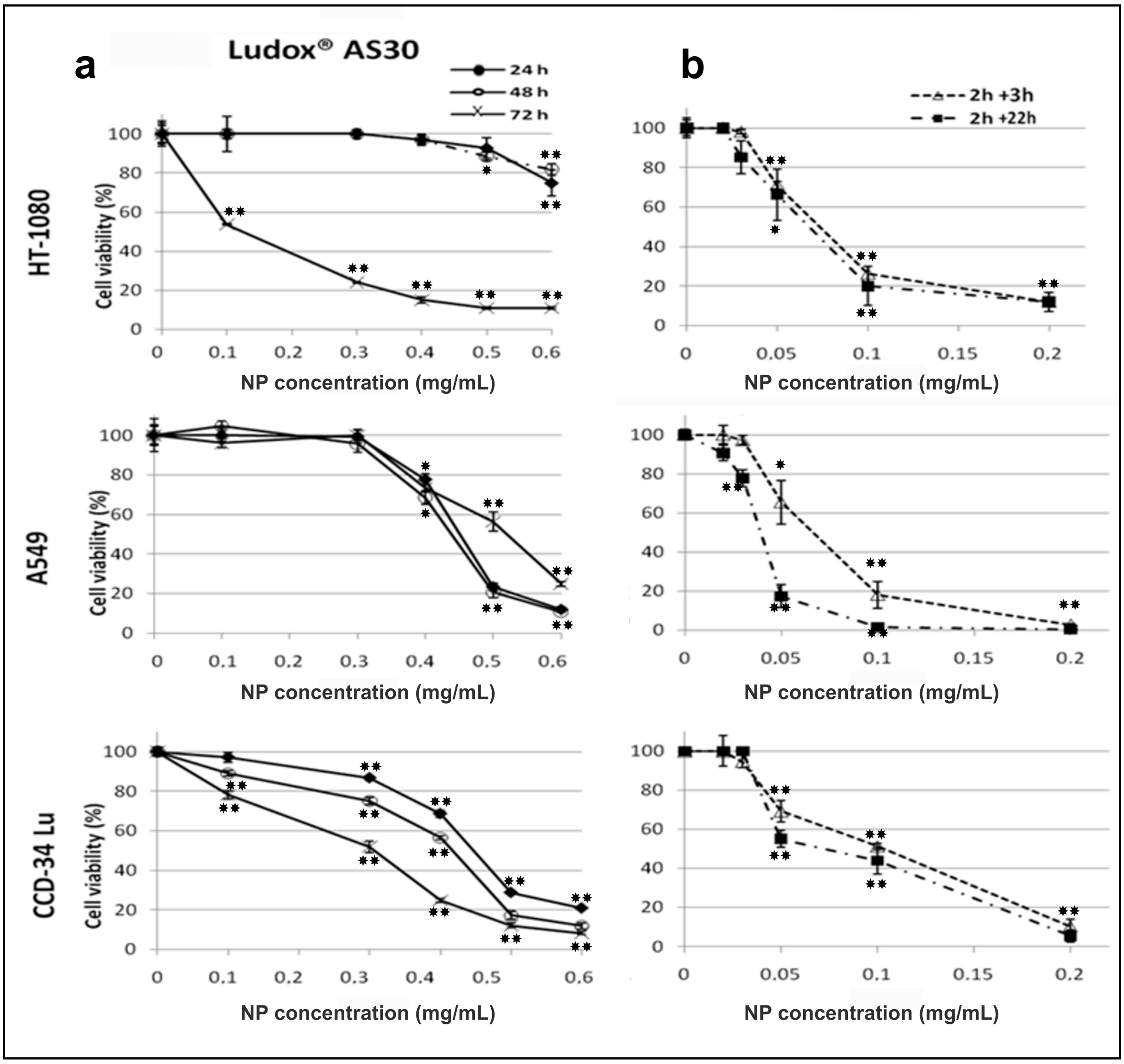

Fig.S3. Cell survival measured by clonogenic assay in HT-1080, A549 and CCD-34Lu cells treated with increasing concentrations of Ludox® NPs AS30. Cell cloning was performed after a 24h treatment with NPs in medium containing 3% of serum (a), or after a 2h treatment in serum-free medium followed by a recovery for 3 or 22h in complete medium (b). The data represent mean ± S.D. ($3 \leq n \leq 12$). *$p < 0.05$, **$p < 0.01$ (t test; treated vs. control cells).

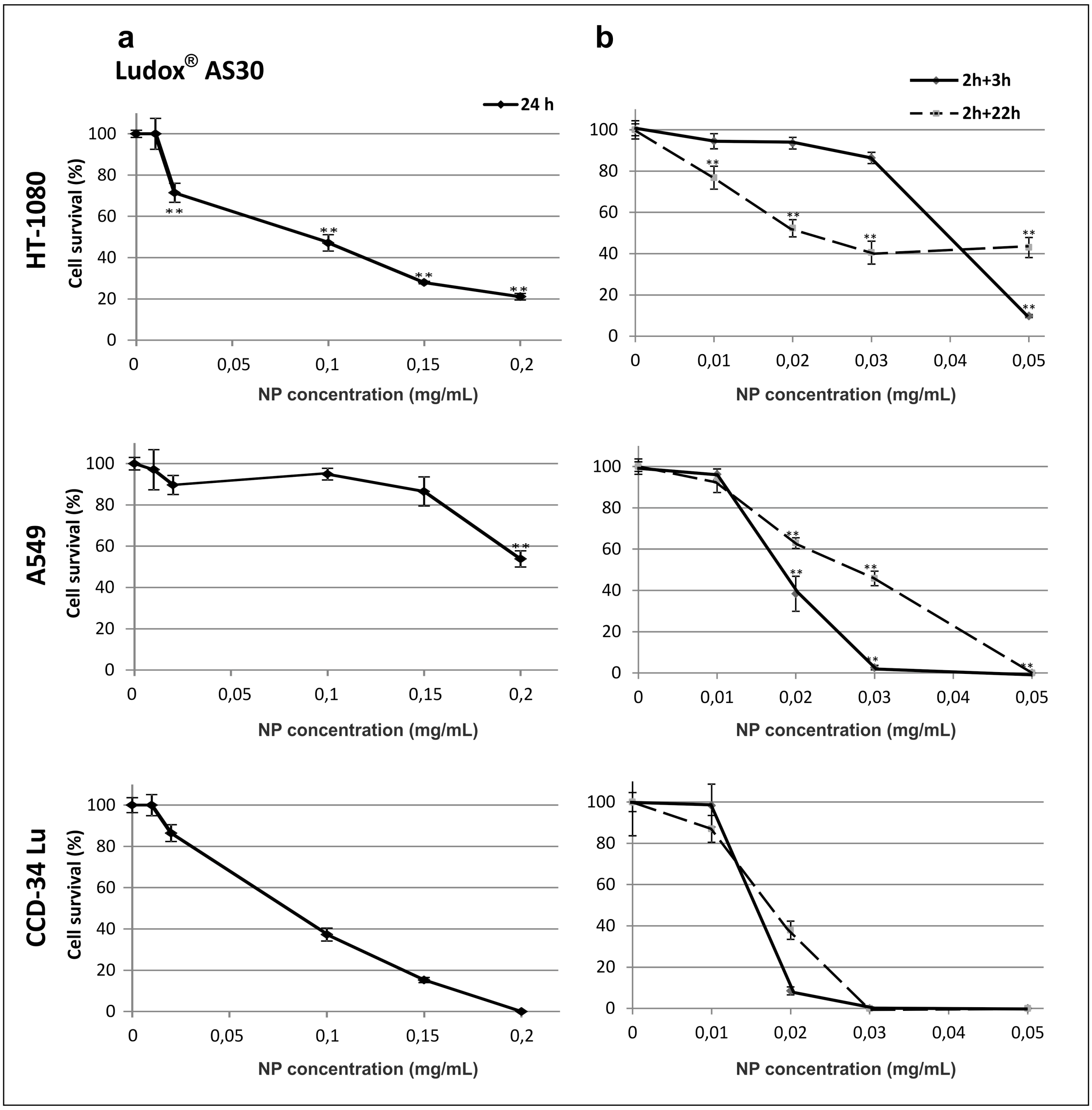

Supplement: Supplementary file 1 — MOESM (PDF 9721 kb) [file 216_2012_6246_MOESM1_ESM.pdf]
